# Predictors of severity and outcome of multiple sclerosis relapses

**DOI:** 10.1186/s12883-023-03109-6

**Published:** 2023-02-13

**Authors:** Hassan Saad Hosny, Hatem Samir Shehata, Sandra Ahmed, Ismail Ramadan, Sarah Sherif Abdo, Amr Mohamed Fouad

**Affiliations:** 1 Neurology Department, Faculty of Medicine, Cairo University, Giza, Egypt; 2 Neurology Department, Faculty of Medicine, Alexandria University, Alexandria, Egypt

**Keywords:** Multiple sclerosis, Prognosis, Recovery, Attack, Relapse, Severity, Outcome, Tempo, Prodrome

## Abstract

**Background:**

Multiple Sclerosis (MS) is a demyelinating disease of the central nervous system (CNS). The most common type of MS is the relapsing–remitting MS (RRMS) where relapses are the main component of the disease course. However, the relationship between the characteristics of the relapses on one hand and their severity and outcome on the other hand has not been fully characterized.

**Objectives:**

To explore the characteristics of relapses among a cohort of Egyptian MS patients and their relation to the severity and outcome of the disease.

**Subjects and methods:**

We analyzed 300 attacks from 223 patients in a retrospective study to identify demographic, clinical and paraclinical (laboratory and radiological) factors affecting: 1- Severity of relapses (the difference between the EDSS at the day of maximum worsening and the EDSS before the onset of the attack). 2- Outcome of relapses (the difference between the EDSS at the day of maximum improvement and the EDSS before the onset of the relapse).

**Results:**

Severe attacks were most likely to occur in patients who are males, single, presenting with poly-symptomatic presentation, slower tempo of evolution of attack symptoms, longer duration of the attack**,** absence of DMTs at the time of the attack. The risk of having a severe relapse is more than 3 times when the patient is single. Regarding attack outcome, poorly recovered attacks were more common in patients with older age at disease onset and at attack onset, male sex, higher number of relapses, longer duration of illness prior to the attack, severe relapses, polysymptomatic presentation, associated cognitive symptoms, slower tempo of symptom evolution, longer duration of the attack, patients on OCPs, smoking, and presence of black holes in brain MRI. The risk of having relapses with partial or no recovery is more than five times when the patient has black holes in brain MRI and more than 4 times when the patient is a smoker.

**Conclusion:**

Bearing in mind the demographic characteristics as well as the clinical and paraclinical characteristics of each attack and their relation to attack severity and outcome are a key to understanding the individual disease course of every patient and hence tailoring the best therapeutic plan suitable for his individual needs. In other words, prompt, rapid intervention in male patients, polysymptomatic attacks, slower tempo of evolution of attack symptoms and longer duration of the attack should be adopted since these factors are predictive of severe relapses as well as poor relapse outcome.

## Introduction

Multiple sclerosis (MS) is the most common demyelinating disease of the central nervous system in young adults [1].

The two main MS phenotypes are the relapsing and the progressive disease phenotypes [2]. Both could occur throughout the disease course in MS [3]. The relapsing disease course is characterized by the occurrence of clearly defined relapses/attacks which are defined as acute or subacute neurological deficits of at least 24-h duration and occurring at least 30 days apart from a previous attack and not attributed to worsening in the context of concomitant infection or fever. These attacks most commonly tend to remit either completely or partially leaving a residual deficit and hence the term Relapsing Remitting MS (RRMS) which is the most common type [4].

On the other hand, the progressive disease course is characterized by progressive accumulation of disability with occasional plateaus and minor improvements and even minor exacerbations but without precise specific onsets and with no obvious improvements or remissions [5].

Symptoms of MS relapse typically develop gradually over hours to days, reaching a in some of days, followed by a gradual and variable recovery course over weeks to months. Both hyperacute and very gradual (e.g., > 12 weeks) presentations are unusual and should point to alternative etiologies [6].

Confirmation of MS relapse can sometimes be challenging for several reasons. First, patients may underreport or overreport their symptoms. In one survey, about 28% of patients did not report their most recent relapse [7].To the best of our knowledge, no simple biomarker or algorithm is available to confirm an MS relapse, and its diagnosis mainly depends on clinical judgment.

The unpredictability about the future of disease course has always been of great concern to the patient as well as the doctor. It is worrisome to all patients, even those with low disability. Prognostic factors are also crucial for clinicians at an early stage of the disease to balance the cost benefit of early immunomodulatory and aggressive therapies bearing in mind the potential adverse effects versus the predicted disease course.

## Subjects and methods

This study includes a retrospective analysis of 300 attacks from 223 patients recruited from the multiple sclerosis (MS) unit in Kasr Alainy, Cairo University Hospitals in the period between May 2020 and November 2021 and fulfilling the diagnosis of relapsing remitting multiple sclerosis (RRMS) as defined by the 2017 revised McDonald’s Criteria [4], with age > 16 years and presenting with an acute neurological deficit defined as new or worsening neurologic symptoms in the setting of a presumed relapse.

The following data about each relapse included in the study were collected from all the patients:

A) Demographic information and information regarding attack-related circumstances as: age at disease onset, age at attack onset, sex, marital status, family history of MS or autoimmune disease, total number of relapses and duration of disease prior to the attack (months).
B) Attack-related clinical data:
  1- Attack definition: Attack is defined as an acute or subacute neurological deficit which could either be a new symptom or a recurrence or worsening of an already existing neurological deficit attributable to MS. The attack should exceed 24 h after a period of at least 30 days of disease stability [8].
    Deterioration of already existing neurological symptoms due to fever or concomitant infection (pseudo-exacerbation) was not considered an attack. Symptoms occurring within 30 days of the initial presentation of an attack were considered as part of the same attack [8].
  2-Attacks were either first-ever attacks or attacks occurring during the course of the disease.
  3-Presentation of attacks were either Mono-symptomatic or poly-symptomatic.
  4-Time to maximum worsening (Zenith level) (in days), time to maximum improvement:
    The tempo of worsening of the attack was estimated by collecting the data regarding the date of onset, the number of days till maximum worsening (zenith), the number of days till the start of improvement and till maximum improvement after which patients reported no further subjective improvement [8].
    Function score (FS) scores as well as total extended disability status scale (EDSS) score were calculated at onset, at the zenith and at the first follow-up visit after the day of maximum improvement [8].
  5- Duration of the attack (in days): the time between the initial symptoms of the attack -defined by the date of onset- and the date of maximum improvement. It was subdivided into short (≤ 30 days), intermediate (31–60 days), and long (> 60 days) [8].
  6- Severity of the attack: Severity was estimated by calculating the difference between the EDSS score at the day of maximum symptom worsening and the EDSS score before the onset of the attack. It was further subdivided into very mild or mild (0 or 2 point), moderate (2.5 or 3 points), and severe (3.5 points or more) [8].
  7-The mean total number of relapses is the average number of relapses that the patient has experienced throughout his disease course till the time of data collection for this study (repeated for attacks reported from the same patient).
  8-Associated depression, fatigue and cognitive symptoms were identified.
  9-Precipitating factors: pregnancy, post-partum period, oral contraceptive pills (OCPs) and hormonal therapy as part of assisted reproductive techniques (ARTs), psychosocial stressors and preceding infection as well as smoking.
  10-MS Prodromal Symptoms: MS prodromal symptoms were viewed in two contexts; first, in the context of symptoms in the 5 to 10 years prior to first demyelinating event and MS diagnosis and this was evaluated by history taken from the patients regarding multiple hospital or physician visits for non-specific symptoms as well as patient records and registry data [9].
    Second, in the context of their association with each attack and were defined as early symptoms or a constellation of symptoms\signs that preceded the diagnosable symptoms\signs of the attack. In this context, the prodromal manifestations were explored on a scale of days to weeks prior to the attack.
  11-Assessment of the outcome of relapses: for each FS we calculated the difference between the score at the time of maximum improvement and the score before the onset of the relapse (0 for the first attack); accordingly, relapses were divided into 2 categories; the full or almost full recovery group with EDSS returning back to baseline with a maximum increase by 1 point compared to the baseline (*n* = 204), and the partial or no recovery group on the other hand with EDSS increase of 1.5 points or more compared to the baseline (*n* = 96) [10].
  12-History of attack treatment was classified as IV methylprednisolone or plasma exchange or no treatment at all.
  13- History of DMT intake and compliance before and at the time of the attack were determined.
C) Para-clinical Data:
  1-MRI Brain at time of the attack: Data regarding total number of white matter lesions, site of lesions, presence of contrast enhanced lesions and presence of T1 hypointense lesions (black holes).
  2-MRI Cervical spine at time of attack: Data regarding number of segments involved (< 3\ ≥ 3) and the presence of contrast enhanced lesions.

### Statistical methods

SPSS (statistical package for social sciences) version 18.0 was used for statistical analysis. Mean ± standard deviation represent quantitative variables and median with range when distribution did not follow normality. Number and percentages represented qualitative data and Chi-square and Fischer's exact tested proportion independence. Odds ratio (95% confidence interval) was used to calculate the risk of having severe relapses or poorly recovered relapses. Parametric and non-parametric t-tests were used for comparing mean values of two independent groups. Multivariable logistic regression analysis was used to detect independent variables that determine the probability (expressed as odds ratio & 95% CI) of having severe relapses and poorly recovered relapses. *P* value is always 2 tailed and significant at 0.05 level.

### Ethical statement

The Research Ethics Committee (REC) of Neurology department, Cairo University, approved the study protocol, which followed the principles outlined in Helsinki’s declaration. Informed written consent was taken from all participants and legal guardian(s) of the patients with mental disorders before the enrollment to participate in the study.

## Results

### Descriptive results

#### Demographics data

In our cohort generally, the number of female and male patients enrolled in the study was 162 (72.6%) and 61 (27.4%) respectively with estimated female to male ratio of 3:1. Age at disease onset ranged from 16 to 48 with a mean of 27.3 ± 6.6 and median 26 (IQR 22 to 32), while age at onset of the attack ranged from 16 to 50 with a mean of 28.8 ± 7 and a median 28 (IQR 24 to 32.5).

Regarding marital status, 134 (60%) patients were married and 89 (40%) patients were single. Family history of MS was positive in 18 patients (8.07%), while family history of autoimmune disease was reported in 14 patients.

#### Attack-related clinical data

Out of the 300 attacks included in our study, 169 attacks (56.3%) were first ever attacks and 131 attacks (43.7%) were attacks that occurred during the course of the disease. The duration of the attack ranged from 10 to 90 days with a mean of 40.2 days ± 15.8 days and a median of 40 (IQR: 30 to 50 days). The attack related clinical data are summarized in Table 1.

**Table 1:** The attack related clinical data

		N(%)
Attacks	first ever attacks	169 (56.3)
	attacks that occurred during the course of the disease	131 (43.7)
Presentation	Mono-symptomatic	121 (40.3)
	Poly-symptomatic	179(59.7)
Associated symptoms	Depression	153 (51)
	Fatigue	201 (67)
	Cognitive	92 (30.7)
Precipitating factors	Pregnancy	3 (1)
	Post-partum	15(5)
	OCPs	33(11)
	Assisted reproductive techniques	12(4)
	Psychosocial stressors	107(35.7)
	Preceding infection	7(2.3)
	Smoking	82(27.3)
MS prodromal symptoms were reported by 114 patients (51.12%) 5 to 10 years prior to their first presentation with typical demyelinating symptoms suggestive of MS	Headache or non-specific sensory symptoms	102(89.4)
	Orthopedic consultations for joint pains	64(56.14)
	Psychiatric help for depression or anxiety	22(19.2)
	Dermatological problems as non-specific rash	(11.4)
Prodromal symptoms in relation to the included attacks	Depression and anxiety	41(13.7)
	Neurological symptoms predominantly headache	128(42.7)
Treatment of the attack	IVMP	254(84.7)
	No acute treatment	46(15.3)
	Plasma Exchange	8 (2.7)
Disease modifying therapy intake at time of attack	Yes	78(26)
	No	222(74)
Severity	Mild to Moderate	183(61)
	Severe	117(39)
Outcome	Full or almost full recovery	204(68)
	Partial or no recovery	96 (32)
EDSS	Time of calculation	
	Before the onset	0 (0- 1)
	After the onset	3.5 (3 – 4)
	At the zenith	3.5 (3 – 4.5)
	After recovery	1.5 (1–2)

#### Para-clinical data

Para-clinical test results are shown in Table 2.

**Table 2:** Para-clinical test results

	N(%)
**1-Total number of lesions in MRI brain:**	
< 4	35(11.66)
4 – 9	164(54.67)
≥ 10	101(33.67)
**2-Gadolinium Contrast Enhancement:**	
Positive	26 (8.7)
Negative	229 (76.3)
Not administered	454545454455(15)
**3-Black holes in MRI**	
Present	56(18.7)
Absent	244(81.3)
**4-MRI Cervical spine**	
Positive	214(71.3)
Negative	86(28.7)

### Comparative results

#### Comparison between mild and moderate relapses on one hand and severe relapses on the other hand regarding demographic, clinical and paraclinical characteristics

Severe attacks were more likely to occur in patients who are males, single, presenting with poly-symptomatic presentation, slower tempo of evolution of attack symptoms, longer duration of the attack, absence of DMTs at time of the attack. However, Psychiatric attack prodromal symptoms were more common in the mild to moderate attacks (Tables 3, 4).

**Table 3:** Comparison between Mild, moderate and severe relapses; full recovered and unrecovered relapses regarding different numerical parameters

	Mild + Moderate Relapses (*n* = 183)		Severe relapses (*n* = 117)			Full or almost full recovery (*n* = 204)		Partial or no recovery (*n* = 96)		
	Mean ± Sd	Median(IQR)	Mean ± Sd	Median(IQR)	*P* value	Mean ± Sd	Median(IQR)	Mean ± Sd	Median(IQR)	*P* value
Age at disease onset	26.8 ± 6.2		27.9 ± 7.1		0.17	26.72 ± 6.028		29.52 ± 7.31		***0.0002***
Age at onset of attack	28.5 ± 6.7		29.1 ± 7.3		0.4	27.064 ± 6.22		32.479 ± 7.1		** < ** ***0.0001***
EDSS before the onset		0 (0—1.5)		0 (0 – 0)	***0.0005***		0 (0–0)		1(0 – 2)	** < ** ***0.0001***
EDSS after the onset		3 ( 3–4)		4(3.5 – 4.5)	** < ** ***0.0001***		3(2.5 – 3.5)		4 (3.5 – 4.5)	** < ** ***0.0001***
Time to maximum worsening (Zenith level)(days)		1(1 -2)		2(1 – 3)	** < ** ***0.0001***		1(1–2)		2(1–3)	***0.005***
EDSS at the Zenith Level		3 (2.5 – 3)		4.5 (3.5 – 6)	** < ** ***0.0001***		3.5 (3 – 4)		4 (3.5—6)	** < ** ***0.0001***
Time to maximum improvement / Duration of the attack (days)		30(25- 45)		50(35 – 60)	** < ** ***0.0001***		30(25–50)		50(40–60)	** < ** ***0.0001***
EDSS after recovery		1 (1- 2)		2 (1.5 – 2.5)	** < ** ***0.0001***		1 (1 – 2)		2.5 (2 – 3)	** < ** ***0.0001***
Total number of relapses		3(2–4)		2 (1 -3)	***0.005***		2(1–3)		3(2–4)	** < ** ***0.0001***
Duration of disease prior to the attack (months)		6 (0–30.5)		0 (0–10)	0.07		0(0–7)		16(0–60)	** < ** ***0.0001***

**Table 4:** Comparison between Mild, moderate and severe relapses; full recovered and unrecovered relapses regarding different categorical parameters

	Mild + Moderate Relapses (*n* = 183)	Severe relapses (*n* = 117)	***P Value***	Full or almost full recovery (*n* = 204)	Partial or no recovery (*n* = 96)	***P Value***
	N(%)	N(%)		N(%)	N(%)	
Sex						
Males	35 (19.1)	42 (35.9)	***0.001***	45(22.1)	32(33.3)	***0.03***
Females	148 (80.9)	75 (64.1)		159(77.9)	64(66.7)	
Marital status						
Single	61(33.3)	55(47)	***0.01***	86(42.2)	***30(31.2)***	0.07
Married	122(66.7)	62(53)		118(57.8)	66(68.7)	
Outcome						
Partial or no recovery	43(23.5)	53 (45.3)	** < ** ***0.001***			
Full or almost full recovery	140 (76.5)	64 (54.7)				
Severity						
Mild + Moderate				140(68.6)	43(44.8)	** < ** ***0.0001***
Severe				64(31.4)	53(55.2)	
Symptomatic presentation						
Mono	118 (64.50	3(2.6)	** < ** ***0.001***	96(47.1)	25(26)	***0.0005***
Poly	65 (35.5)	114(97.4)		108(52.9)	71(74)	
Associated						
Fatigue	124(67.8)	77(65.8)	0.7	128 (62.7)	73 (76)	0.2
Depression	93(50.8)	60(51.3)	0.9	107 (52.5)	46 (47.9)	0.4
Cognition	67(36.6)	25(21.4)	***0.005***	51(25)	40(41.7)	***0.001***
Precipitating factor						
Post-partum relapse ^a^	11(7.4)	4(5.3)	0.7	10(6.3)	5(7.8)	0.7
contraception ^a^	24(16.2)	9(12)	0.4	16(10.1)	17(26.6)	***0.003***
Assisted reproductive technique ^a^	7(4.7)	5(6.7)	0.7	7(4.4)	5(7.8)	0.3
Preceding infection	2(1.1)	5(4.3)	0.07	4(2)	3(3.1)	0.5
Psychosocial stressors	60(32.8)	47(40.2)	0.1	80(39.2)	27(28.1)	0.06
Smoking	43(23.5)	39(33.3)	0.06	44(21.6)	38(39.6)	***0.001***
Prodromal symptoms						
Neurological	73(39.9)	55(47.0)	0.2	94 (46.1)	34 (35.4)	0.08
Psychiatric	33(18.0)	8(6.8)	***0.005***	24 (11.8)	17 (17.7)	0.1
Treatment of attack						
IVMP	147(80.3)	107(91.5)	***0.009***	169(82.8)	85(88.5)	0.2
PLEX	0(0)	8(6.8)	***0.0002***	0(0)	8(6.8)	***0.0002***
DMT intake at time of the attack	61(33.3)	17(14.5)	***0.0002***	36(17.6)	42(43.7)	** < ** ***0.0001***
Black holes	33 (18)	24(20.5)	0.6	20(9.8)	36(37.5)	** < ** ***0.0001***
MRI cervical spine						
normal	53 (29)	83 (71)	** < ** ***0.0001***	45 (22.1)	41 (42.7)	***0.0009***
< 3 segment	120(65.6)	32(27.3)		151(74)	51(53.1)	
> 3 segment	10 (5.4)	2 (1.7)		8(3.9)	4(4.2)	

^a^ Analysis for female patients only

#### Comparison between attacks with favorable outcome vs attacks with poor outcome regarding demographic, clinical and paraclinical characteristics

Regarding attack outcome, poorly recovered attacks were more common in patients with older age at disease onset and older age at attack onset, male sex, higher number of relapses, longer duration of illness prior to the attack, severe relapses, polysymptomatic presentation, associated cognitive symptoms, slower tempo of symptom evolution, longer duration of attack, patients on OCPs, smoking, and presence of black holes in brain MRI scans (Tables 3, 4).

Associated fatigue and depression as well as neurological and psychiatric prodromal symptoms showed no significant difference between the full or almost full recovery group and the partial or no recovery group.

### Regression analysis

Results of multivariable logistic regression analysis for prediction of having a severe attack (the utilized variables were; total number of relapses, EDSS before attack onset (baseline EDSS), gender, marital status, use of DMT, mono and poly-symptomatic presentations, associated cognitive complaints, presence of prodromal psychiatric symptoms, and results of MRI cervical spine) showed a significant regression, *p* < *0.0001* and R2 = 0.54. The risk of having a severe relapse is more than triple when the patient is single (not married) (OR = 3.72, CI = 1.5 – 8.8, *p* value = *0.002*). Similar results were found regarding EDSS before attack onset (baseline EDSS), total number of relapses and presence of psychiatric prodromal symptoms (Table 5).

**Table 5:** Results of multivariate logistic regression analysis (using stepwise model) for prediction of having a severe attack (table showing only the significant results)

Variable	Odds ratio	95% CI	*P* value	Relative risk	95% CI	*P* value	NNT ^a^	95% CI
EDSS before relapse onset (Baseline EDSS)	0.272	0.154 to 0.479	** < ** ***0.0001***	-	-	-	-	-
Single (marital status)	3.729	1.568 to 8.87	***0.003***	1.41	1.06 -1.86	***0.01***	7.313 (Harm)	40.27 (Harm) to 4.02 (Harm)
Presence of psychiatric prodromal symptoms	0.219	0.074 to 0.65	***0.006***	0.379	0.18—0.79	***0.009***	8.932 (Benefit)	5.25 (Benefit) to 30.05 (Benefit)
Total number of relapses	0.789	0.66 to 0.94	***0.007***	**-**	**-**	**-**	**-**	**-**

^a^ Number need to treat

Results of multivariable logistic regression analysis for prediction of having relapses with partial or no recovery (the utilized variables were**;** total number of relapses, EDSS after onset, gender, marital status, age at onset of disease, smoking, total number of lesions and the presence of black holes in MRI brain, severity and duration of attack, duration of disease before attack, use of DMT, mono and poly-symptomatic presentations, associated cognitive symptoms, prodromal symptoms and results of MRI cervical spine) showed a significant regression, *p* < *0.0001* and R2 = 0.47, the risk of having relapses with partial or no recovery is more than five times when the patient has black holes in MRI and more than 4 times when the patient is a smoker ( OR = 5.425; 4, CI = 1.401 – 21; 1.58 – 10.1; *p* value = *0.014, 0.003* respectively), similar results were found regarding EDSS after onset,total number of relapses, duration of the attack, duration of disease before the attack, age at onset (Table 6).

**Table 6:** Results of multivariate logistic regression analysis (using stepwise model) for prediction of having attacks with partial or no recovery (table showing only the significant results)

Variable	Odds ratio	95% CI	*P*	Relative risk	95% CI	*P* value	NNT ^a^	95% CI
Presence of black holes in brain MRI	5.425	1.401 to 21.008	***0.014***	3.825	2.343 – 6.242	** < ** ***0.0001***	3.611 (Harm)	5.325(Harm) -2.731 (Harm)
Duration of Attack	1.04	1.015 to 1.077	***0.003***					
Duration of Disease before the attack	1.030	1.005 to 1.055	***0.016***					
EDSS after onset	1.532	1.054 to 2.227	***0.025***					
Age at onset of disease	1.1774	1.104 to 1.254	** < ** ***0.0001***					
Presence of smoking	4.0007	1.583 to 10.107	***0.0034***	1.835	1.28—2.63	***0.0009***	5.551 (Harm)	13.518(Harm) to 3.493 (Harm)
Total Number of relapses	3.0493	2.09 to 4.429	** < ** ***0.0001***					

^a^ Number need to treat

## Discussion

Multiple sclerosis is considered the most common inflammatory condition affecting the CNS [11]. Relapse severity and residual disability are two concerns that are most worrisome to the patient as well as to the treating neurologist. Patients are always worried about the outcome of their relapses and any upcoming events, and their neurologists cannot give them accurate expectations in many situations; however, the neurologists’ personal expertise may sometimes help [12]. Although relapses are often a concern by themselves, patients are often placed on treatment quite early after diagnosis. Relapses, particularly in regards to severity and disability, typically indicate ineffective treatment and the need to change treatment (such as with second-line).

Our study evaluated the factors affecting relapse severity and recovery as the two most important outcomes sought by the patient and the neurologist during the management of a multiple sclerosis relapse. 300 relapses from 223 RRMS patients were the subject of this study.

Relapses were more likely to be severe in male patients. These findings go in parallel with previous studies, which showed that relapses tend to be mild to moderate in female patients, whereas the majority of relapses that occur in male patients tend to be severe [13–15].

Relapses occurring in single patients tended to be severe as well. This was also evident in the study done by Abbasi and colleagues, which showed that although most of the patients in their study were married, the severity of disease in this group was less than in the single patients’ group [16]. These findings show the possible positive effect of stable relationships on the course of disease, highlighting the role of the patient’s support system.

Regarding the effect of disease-modifying therapies (DMTs), our results showed that patients not on DMTs were more likely to experience severe attacks. This is in agreement with previous studies done by Naldi [8], Noyes, & Weinstock-Guttman [17], Traboulsee and colleagues [18], and Kantarci and colleagues [19]. The latter study showed that patients presenting with unfavorable outcomes after the initial relapse could benefit from immediate DMT initiation for a more ambulatory-benign disease course.

The findings that attacks with polysymptomatic presentations were more likely to be severe agree with previous work done by Naldi as well as Baghizadeh and colleagues [8, 20].

Regarding attack outcomes and in agreement with our results, Ribbons and colleagues compared the difference in disability and progression between males and females by estimating the time to reach EDSS milestones 3 and 6, as well as the time to develop secondary progressive MS. They found that males showed significantly faster progression regarding EDSS than females, and females had a lower risk of developing secondary progressive MS [21].

Our study showed that older patients were more likely to have an unfavorable outcome, which is in agreement with Cossburn and colleagues, who stated that the ability to recover from an initial relapse decreases with age, suggesting that the accumulation of disability in MS is an age-dependent response to relapse [22]. Furthermore, Conway and colleagues studied age as a critical determinant in recovery from multiple sclerosis relapses and found that patients with RRMS are more likely to lose recovery potential linearly as they age [23].

According to our results, the risk of having an unrecovered or partially recovered attack is more than four times higher if the patient is a smoker. In the systematic review conducted by Hempel and colleagues, the role of tobacco smoking in poor physical MS outcomes was demonstrated. They showed that the risk of converting to secondary progressive MS is 55% greater for smokers than non-smokers [24]. Similar results were also demonstrated in another study done by Mckay and colleagues [25]. In light of these findings, we would encourage our patients to give up smoking for better relapse outcomes and overall health.

Our study showed that the more relapses, the worse the outcomes of the relapse. This is in agreement with a previous study, which showed that the median time from the onset of multiple sclerosis to reaching an EDSS score of 4, 6, and 7 was significantly affected by the degree of recovery from the first relapse, time to a second neurological episode, and the number of relapses in the first 5 years of the disease [26]. The same findings were evident in other studies done by Goodin and Sevim as well as Koch-Henriksen and colleagues [27–29]. Similar findings regarding the duration of illness prior to the attack were also evident; the longer the disease duration, the worse the outcome of relapse. This is consistent with the findings of a study conducted by Kalincik and colleagues, who discovered that relapses with higher impact and poorer recovery were associated with a longer MS duration [30]. It also agrees with other studies done by Stewart and colleagues as well as Briggs and colleagues [31, 32].

Regarding the attack outcome and mono – or polysymptomatic presentation, our study showed that the polysymptomatic presentations were more likely to have a worse outcome. This is in agreement with other studies done by Leone and colleagues, Sevim, as well as West which showed that polysymptomatic presentations are predictors of incomplete recovery from relapses in multiple sclerosis [10, 33, 28].

Our study showed that black holes in brain MRI scans increased the risk of poor recovery from attacks by five times. This is in agreement with the study done by Zivadinov and Leist, which suggested that T1 hypointense lesions correlated better with outcome and disability than T2 lesions [34]. Additionally, another study done by Truyen and colleagues speculated that accumulation of T1 lesions could be the MR equivalent of failure of remission [35].

In our cohort, patients reported prodromal symptoms (MS prodrome) prior to their first presentation with MS, which were mostly psychiatric symptoms such as anxiety and depression. These psychiatric prodromal symptoms could affect the severity of relapses. This is in line with the study done by Vienažindytė and colleagues, which hypothesized that detecting prodromal symptoms as early as possible could play an important role in identifying the course of the MS and predicting disability progression [36].

There are two major limitations in this study that could be addressed in future research. First, we relied on the EDSS as an outcome indicator to assess the severity and recovery of relapses. Since the EDSS is an ordinal rating scale, a 1-point difference in one part of the scale does not represent the same 1-point difference in another part of the scale, thus sometimes leading to misinterpretation of results. Additionally, at higher EDSS scores, the scale depends mainly on ambulation, so only relapses affecting the motor sub-score can change the scale. Second, we were unable to include all relapses in the records because of missing data in some of them or incomplete recall of events during those relapses when interviewing the patients.

To conclude, our study provides new insights into the most frequently encountered questions neurologists face after the diagnosis of MS: how to predict attack severity and outcome? In our cohort, we tried to explore all factors affecting these outcomes, providing adequate knowledge and reassurance to patients as well as timely management accordingly. Considering the large number of variables examined, the results should be considered from an exploratory perspective.

## Data Availability

The datasets used and/or analyzed during the current study are available with the corresponding author (Amr M Fouad) upon request.

## Abbreviations

MS: Multiple sclerosis
CNS: Central nervous system
RRMS: Relapsing remitting multiple sclerosis
EDSS: Expanded Disability Status Scale
DMT: Disease modifying therapy
MRI: Magnetic resonance imaging
FS: Fuctio system
SPSS: Statistical package for social sciences
SD: Standard deviation
IQR: Interquartile range
